# Oxidative and Nitrosative Stress in Major Depressive Disorder: A Case Control Study

**DOI:** 10.3390/brainsci12020144

**Published:** 2022-01-21

**Authors:** Aditya Somani, Abhishek Kumar Singh, Bandna Gupta, Sheela Nagarkoti, Pronob Kumar Dalal, Madhu Dikshit

**Affiliations:** 1 Department of Psychiatry, King George’s Medical University, Lucknow 226003, India; dr.adityasomani@gmail.com (A.S.); drbandna@yahoo.co.in (B.G.); docpkdalal@gmail.com (P.K.D.); 2 Pharmacology Division, CSIR-Central Drug Research Institute, Lucknow 226031, India; singh82biotech@gmail.com (A.K.S.); sheelanagarkoti@gmail.com (S.N.)

**Keywords:** depression, nitric oxide, nNOS, oxidative stress, neutrophils, first-degree relatives

## Abstract

Introduction: The role of increased oxidative stress and alterations to the nitric oxide (NO) pathway have been implicated in major depressive disorder (MDD). The two pathways interact closely with each other but have not been studied simultaneously in MDD. This study aimed to assess and compare the levels of oxidative and nitrosative stress in the neutrophils (PMNs) of drug-naive MDD patients and their first-degree relatives. Methods: 29 drug-naive MDD patients and 27 healthy first-degree relatives and healthy controls aged 18–45 years were included in this study. An assessment of the levels of reactive oxygen species (ROS), nitrites, neuronal NO synthase (nNOS), and myeloperoxidase in PMNs, and cortisol in serum was carried out. Results: Compared to healthy controls, the generation of free radicals, myeloperoxidase activity, and nNOS mRNA expression in PMNs, and cortisol level in serum were significantly higher in drug-naive depression patients. Indeed, increased levels of myeloperoxidase and serum cortisol were also noted in first-degree relatives. The total nitrite content in the PMNs and plasma however was significantly lower in both patients and first-degree relatives. Interestingly, a positive correlation was established in the ROS levels in the PMNs, plasma and neutrophil nitrite, and the serum cortisol level between MDD patients and their first-degree relatives. Conclusion: The results of this study contribute towards a better understanding of the familial association of depressive disorder, and demonstrate for the first time that neutrophil ROS/RNS, plasma nitrite, and serum cortisol levels are positively correlated between MDD patients and their first-degree relatives. However, further studies in larger, more diverse samples are needed to extend these pathways as potential biomarkers to identify persons at high risk for psychopathology at an early stage.

## 1. Introduction

Depression is a common mental disorder that affects people all over the world. According to the World Health Organization, it currently affects nearly 264 million people globally across all age groups [1] and is projected to become the second leading cause of worldwide disease and disability by 2030 [2]. Compelling evidence indicates that severe depressive disorder could lead to suicide [1]. The etiopathogenesis of depression is believed to be multifactorial and involves a close interplay of biological and psychosocial factors [3]. For a long time, the theories and research in relation to the biological factors leading to depression were largely concentrated around the monoamine neurotransmitters i.e., norepinephrine, dopamine, serotonin, and histamine. Of late, the search for other biological factors for depression has expanded and valuable information has been generated regarding the role of other neurotransmitters, second messengers, hormonal regulation, immunological disturbances, and inflammation [3]. An important contender among the possible biological factors leading to depression is oxidative stress [4,5,6,7].

The human brain is believed to be more vulnerable to oxidative stress compared to other organs and tissues in the body [8]. This could be due to the high consumption of oxygen in the brain, which accounts for about 20% of the total amount of oxygen in the body. This vulnerability is further increased by the presence of higher amounts of polyunsaturated fatty acids, iron, and low concentrations of antioxidant enzymes [8]. Several preclinical and clinical studies have shown a positive correlation between the severity of depression and oxidative stress [4,9,10,11,12]. Consequent to increased reactive oxygen species (ROS) levels, depression is also related to depleted levels of antioxidants such as vitamin E, zinc, coenzyme Q10, and glutathione as well as the exhaustion of antioxidative enzyme activities such as xanthine oxidase and superoxide dismutase [4,5,9,10]. Studies in post-mortem brain samples have further displayed increased levels of ROS including peroxide and superoxide and altered levels of antioxidant defenses in depression patients [13,14]. This oxidative vulnerability in the depression population results in damage to DNA and RNA, and telomere shortening. Accordingly, antioxidant treatment has been shown to improve psychiatric symptoms in clinical trials [10]. 

People suffering from unipolar (major depressive disorder) or bipolar depression display dysregulated redox signaling. Nitric oxide (NO), a redox-active small molecule, is an important signaling molecule in many neural processes and also exerts both pro- and antioxidant responses. At lower concentrations, this facilitates the generation of free radicals, while a higher concentration of NO acts as a protector of oxygen free radicals [15,16]. NO is also involved in the release of neurotransmitters (including dopamine), neural development, regeneration, the regulation of gene expression, and synaptic plasticity which is believed to be related to learning and memory [17]. The assessment of NO levels in the body is mainly carried out by assessing the levels of its metabolites, nitrite, and nitrate. Studies conducted in clinical and animal models have demonstrated the possible role of NO and its metabolites in depressive disorder [18,19,20,21,22]. 

NO is synthesized from L-arginine in the presence of the nitric oxide synthase (NOS) enzyme via the L-arginine–NO pathway [23]. Three isoforms of the enzyme NOS have been known viz. endothelial NOS (eNOS), neuronal NOS (nNOS), and inducible NOS (iNOS). Among these, the nNOS, found primarily in nervous tissue, has been linked with neuropsychiatric disorders including depression [7,24,25,26]. Previous studies have also assessed the possible role of nNOS in the anti-depressant action of selective serotonin reuptake inhibitors [27,28,29]. Though nNOS is profoundly expressed by neuronal cells, its localization in the human brain and the reduced cell count of paraventricular nNOS positive neurons in depressed patients restrict its assessment in most clinical studies. Many pieces of evidence indicate that, similar to neurons, human neutrophils (PMNs) also express nNOS [30]. The infiltration of neutrophils in the CNS contributes to the development of numerous neurodegenerative disorders, including MDD [31]. Several clinical studies have shown that an alteration in the nNOS–NO pathway in the human brain is also reflected in circulating neutrophils [32,33]. Therefore, as a proxy marker of the nNOS pathway in the brain, in the present study, we investigated nNOS expression in circulating PMNs of depressive patients. 

The activity of the nNOS is affected by many factors viz. age, gender, diet, diurnal variation, glucocorticoids, antidepressant drugs, nicotine and other substances of abuse, high blood glucose, high cholesterol, deranged liver functions, and major physical illnesses especially autoimmune disorders [4,9,34,35,36,37,38,39]. Any study that plans to assess nNOS would be required to take note of these factors, especially glucocorticoids. The level of serum cortisol is known to be elevated in patients with depression and this resolves with treatment [3]. At the same time, it is also known that patients with depression have elevated levels of inflammatory markers [3,40,41]. Therefore, when planning to assess the oxidative stress and NO pathway in patients of depression, it is imperative to assess a suitable marker of inflammation as well. Myeloperoxidase (MPO) is one such marker that has been assessed in patients with depression and has been found to be positively correlated with the severity of depression [41]. Indeed, lower levels of MPO are linked with reduced cardiovascular diseases, cytokine production, and inflammation that usually coexist with depression [42]. 

To assess the genetic underpinning of these parameters, the assessment of asymptomatic blood relatives of the patients can bring valuable insights to the topic. With these issues in mind, the current study was planned to evaluate the oxidative stress and markers of the NO pathway in patients with depression. The primary aim of this study was to assess and compare the levels of oxidative and nitrosative stress in drug-naive patients (in the current episode) with major depressive disorder (MDD). This study further assessed the association between oxidative/nitrosative stress and the clinical profile of depression patients and its correlation with their first-degree relatives.

## 2. Materials and Methods

### 2.1. Study Setting and Selection Criteria for Participants

The participants for this study were recruited from patients and caregivers attending the outdoor and indoor sections of the department of Psychiatry, King George’s Medical University, Lucknow, India. The method of sampling was convenience sampling. Patients who were diagnosed with MDD as per the *Diagnostic and Statistical Manual of Mental Disorders, 4th edition*, text revision (DSM-IV-TR) [43] were included if they were aged between 18 and 45 years and had not received psychotropic medications or electroconvulsive therapy during the last three months. They were excluded if they were suffering from any other comorbid psychiatric disorder. Among the available first-degree relatives of the patient, anyone was included in the study randomly if he/she was aged between 18 and 45 years and scored less than three on the General Health Questionnaire, 12 item version (GHQ-12) comprising six items that are positive descriptions of mood states (e.g., “felt able to overcome difficulties”) and six items that are negative descriptions of mood states (e.g., “felt like a worthless person”) [44]. First-degree relatives were not included if they suffered from any psychiatric disorder. Healthy controls were included if they were aged between 18 and 45 years and scored less than three on the GHQ-12 [44]. Healthy controls were excluded if they or any of their first-degree relatives suffered from any psychiatric disorder. A validated Hindi translation of the GHQ-12 was used and it was filled in by the participants themselves. The first author (A.S.) assisted the participants if they sought any clarification. Permission for the use of the GHQ-12 in this research was taken from the copyright holders. All the patients and the other subjects were included in the study after taking written voluntary informed consent. The patients and other subjects were excluded from the study if they were suffering from or were taking treatment for any major medical illness, had deranged blood cholesterol/sugar or liver function tests, used substances other than nicotine, or were unable to provide informed consent. 

### 2.2. Procedure

Prior to inclusion in the study, the patients, their relatives, and healthy controls were assessed by a trainee psychiatrist. Thereafter, the diagnosis and suitability for inclusion in the study were confirmed by a qualified psychiatrist. Their clinical details were noted. The severity of depression was assessed with the 17-item Hamilton Depression Rating Scale (HAM-D) [45] and functioning was assessed on the Global Assessment of Functioning (GAF) scale [46]. The study protocol was approved by King George’s Medical University (KGMU) and the CSIR-Central Drug Research Institute, Lucknow, and was conducted in accordance with the Declaration of Helsinki. The study was carried out after approval from the Institutional Ethics Committee, King George’s Medical University, Lucknow, India. The study was conducted from July 2012 to August 2013. 

Blood samples were collected from all the participants after overnight fasting between 8:00 a.m. and 10:00 a.m. The samples were subjected to an assessment of blood sugar, liver function tests, and cholesterol level in the laboratory of Pathology Department at King George’s Medical University, Lucknow, India. If these reports were normal, the samples were further processed at the Laboratory of Pharmacology Division, Central Drug Research Institute, Lucknow, India. 

### 2.3. Separation of Neutrophils and Plasma

Neutrophils were isolated from the venous blood samples collected in sodium citrate (0.129 M, pH 6.5, 9:1-*v*/*v*). Platelet rich plasma was removed by centrifugation at 250× *g* for 20 min at 20 °C (Sigma centrifuge, Roedermark, Germany) and the buffy coat was subjected to a Percoll density gradient at 700 g for 30 min at 25 °C. The neutrophils from healthy volunteers, patients, and their first-degree relatives were isolated as described previously [47], and the purity was ascertained by a Flow cytometer (FACS Calibur, Becton Dickinson, NJ, USA) using a CD15 antibody and it was always more than 95%. The viability of the isolated PMNs was more than 95%, as assessed by the Trypan blue exclusion test [47].

### 2.4. Assessment of ROS Generation 

The relative levels of intracellular ROS were measured using the redox-sensitive probes dichlorodihydrofluorescein diacetate (DCF-DA). PMNs (1 × 10^5^), were incubated with DCF-DA (10 µM) for 15 min at 37 °C and were assessed for ROS generation by acquiring 10,000 events on the FACS Calibur (Becton Dickinson, NJ, USA) and subsequently analyzed by the Cell Quest program (Becton Dickinson, NJ, USA) [48].

### 2.5. Estimation of Neutrophil and Plasma Nitrites

NO production in the plasma or PMNs was determined by measuring the nitrite content, the metabolic end product of NO formation, using the Griess reagent. To estimate the total nitrite content in plasma, cadmium pellets (Sigma, St. Louis, MO, USA) were added to reduce nitrate to nitrite as described earlier [49] and an equal volume of Griess reagent (0.1% aqueous N-(l-naphthyl)-ethylenediamine dihydrochloride, 1% sulfanilamide, and 2.5% phosphoric acid) was added to the respective samples and incubated for 30 min at 37 °C. Nitrite concentration was estimated by measuring the absorbance at 545 nm and 630 nm (wavelength correction) against sodium nitrite as a standard using an ELISA plate reader (BioTek Instrument Inc., Winooski, VT, USA) and represented as µMoles/mL [50]. 

To estimate the total nitrite content in the PMNs, the cells were sonicated on ice after adding hypotonic TKM solution (25 mM Tris-HCl pH-7.4, 5 mM KCl, 1 mM MgCl_2_, and 1% NP-40). The cell lysates were deproteinized by adding 96% cold ethanol (1/2 *v*/*v*) for 30 min at 4 °C and centrifuged at 13,000 rpm for 5 min at 4 °C. To the supernatant, cadmium pellets (Sigma) were added to reduce nitrate to nitrite and were similarly treated with an equal volume of Griess reagent. The total nitrite content in the PMNs was reported as µM/10^7^ cells [50].

### 2.6. Estimation of Neutrophil nNOS Expression

Total RNA from human PMNs was isolated using the Tri reagent and cDNA was synthesized by a RevertAid™ H Minus first-strand cDNA synthesis kit (Fermentas Life Sciences, Vilnius, Lithuania) using an oligo (dT) primer. Quantification of nNOS mRNA was performed by a real-time PCR using the Light Cycler instrument (Roche Applied Science, Lewes, UK) with a 2X Maxima SYBR Green RT-PCR Master Mix (Roche Applied Science, Lewes, UK) and nNOS (forward 5′-TCTAACAGGCTGGCAATGAAG-3′, reverse 5′-TCTCTAAGGAAGTGATGGTTGAC-3′) and β-Actin (forward 5’-AACTGGAACGGTGAAGGTG-3’, reverse 5’-CTGTGTGGACTTGGGAGAGG-3′) primers. A three-step cycling protocol (initial denaturation at 95 °C for 10 min, 45 cycles of 15 s denaturation at 95 °C, 20 s annealing at 57 °C, and 20 s extension at 72 °C) was used to amplify the genes. The specificity of the PCR products was determined by a melting curve analysis consisting of 1 cycle: 95 °C for 0 s, 65 °C for 10 s, 95 °C for 0 s, and 1 cooling cycle: 40 °C for 3 min. The relative fold differences between the groups were calculated by using the comparative cycle threshold (2^−ΔΔCp^) method. β-Actin was used as an internal standard to calculate the relative mRNA expression [47,50]. 

### 2.7. Estimation of Neutrophil MPO Activity

PMNs (5 × 10^6^ cells/mL) were freeze-thawed consecutively three times and then sonicated in three cycles of 10 s each at 95 watts (W-385 Heat System, Ultrasonics Inc., Amelia, OH, USA) in ice. Hexadecyl trimethyl ammonium bromide (HTAB, 27 mM) was added to the cell lysate, incubated at 37 °C for 30 min, and centrifuged at 3000× *g* for 20 min at 20 °C. Twenty microliter samples were mixed with a phosphate buffer (Na_2_HPO_4_-50 mM; NaH_2_PO_4_-50 mM pH6.0), o-dianisidine (7.09 mM), and hydrogen peroxide (4.4 mM). Optical density was recorded at 462 nm using an ELISA plate reader (BioTek Instrument Inc., Winooski, VT, USA) and MPO activity has been expressed as µmole/1 × 10^6^ cells/3 min for PMNs using the molar extinction coefficient of oxidized o-dianisidine ε = 10,062 [M × cm]^−1^ [48].

### 2.8. Estimation of the Cortisol Level

Serum cortisol was estimated by using a Cortisol ELISA kit (DRG, New Jersey, USA), as per the manufacturer’s instructions. Briefly, 20 μL of standard or serum samples were added to wells pre-coated with anti-cortisol monoclonal antibodies followed by the addition of 200 μL enzyme conjugate, mixed for 10 s, and incubated for 60 min at room temperature. After washing with a wash buffer, a substrate solution (tetramethylbenzidine) was added to the respective wells and incubated for 15 min at room temperature. The enzymatic reaction was stopped by adding 100 μL of stop solution (0.5 M H_2_SO_4_) and subsequently the plate was read at 450 ± 10 nm using an ELISA plate reader (BioTek Instrument Inc., Winooski, VT, USA). Standards provided in the kit were used for drawing the standard curve and for calculating the absolute cortisol concentration in the sample. The level of cortisol was reported as ng/mL of serum.

### 2.9. Statistical Analysis

Data have been represented as mean ± SEM and the statistical significance was analyzed by a one-way analysis of variance (ANOVA) followed by a post hoc analysis using Tukey’s multiple comparisons test. Pearson’s correlation coefficients *(r*) were performed to determine if there was any correlation between the patients’ profiles and the investigated parameters or between the investigated parameters of the patients and their first-degree relatives. All statistical analyses were performed with the GraphPad Prism 5.0 program (GraphPad Inc., La Jolla, CA, USA) and a *p*-value less than 0.05 was considered statistically significant.

## 3. Results

By the end of the study period, 29 patients with depression, 27 first-degree relatives, and 27 healthy controls were included in the study. The demographic profile of the study participants is shown in Table 1. There were no significant differences in age and gender between depressed patients, first-degree relatives, and controls. The majority of the patients (86.21%) included in the study were diagnosed to be suffering from MDD in the first episode, while the remaining 14% of patients experienced recurrent MDD. The mean duration of the current episode of depression was 3.94 ± 0.5838 months. A family history of mood disorder was present in only five (17%) of the patients. The average score of patients on HAM-D and GAF was 19.86 ± 0.7636 and 34.10 ± 1.905, respectively. The clinical profile of the patients is shown in Table 2.

### 3.1. Oxidative Stress and MPO in the PMNs of Depression Patients and Their First-Degree Relatives

**Figure 1:**
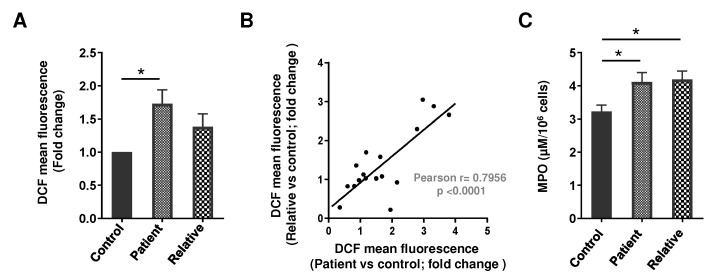
(**A**) Free radical generation (DCF mean fluorescence) in the PMNs of drug-naive depression patients, their first-degree relatives, and healthy volunteers (depression patients *n* = 20, first-degree relatives *n* = 18, healthy controls *n* = 18). DCF mean fluorescence in the PMNs of the healthy control was considered as 1 to calculate the fold change. F (2, 53) = 4.618; * *p* < 0.05. (**B**) Correlation in the ROS levels in the PMNs between patients and first-degree relatives. (**C**) MPO activity in the PMNs of drug-naive depression patients, their first-degree relatives, and healthy volunteers (depression patients *n* = 18, first-degree relatives *n* = 16, healthy controls *n* = 15). F (2, 46) = 4.274; * *p* < 0.05. (**A**,**C**) Data expressed as mean ± SEM.

Free radical generation as assessed by a DCF-DA fluorescent probe was significantly more in the PMNs of drug-naive depression patients (~1.72 fold, *p* < 0.05, 95% CI: −1.305 to −0.1504), as compared to the healthy volunteers (Figure 1). To calculate the risk estimates in the family, we next assessed ROS generation in the first-degree relatives of depression patients. Though no statistically significant change in ROS levels was observed between the PMNs of healthy volunteers and the first-degree relatives, an elevated trend (1.38 ± 0.83-fold) was noted. Furthermore, a positive direct correlation in the ROS levels between patients and first-degree relatives was established (*r* = 0.7956, *p* < 0.0001) (Figure 1A,B, Table 3). On the other hand, no significant interrelation was noted between the ROS generation and the disease severity, HAM-D and GAF score, age, gender, number of episodes, and duration of illness or family history.

MPO is a critical component of the oxidative activity of the neutrophils, so next we measured the MPO activity in these patients. MPO activity was significantly higher in the PMNs of depressive patients (~1.23 fold, *p* < 0.05, 95% CI: −1.759 to −0.02488) and their first-degree relatives (~1.3 fold, *p* < 0.05, 95% CI: −1.856 to −0.07252), compared to healthy volunteers (Figure 1C). In categorizing disease severity in the blood relatives, no correlation was observed. Similarly, MPO activity in the PMNs was independent of disease severity, HAM-D and GAF score, age, gender, number of episodes, duration of illness, and blood relation.

### 3.2. Nitrite Level and NOS Isoform Expression in the PMNs of Depression Patients and First-Degree Relatives

**Figure 2:**
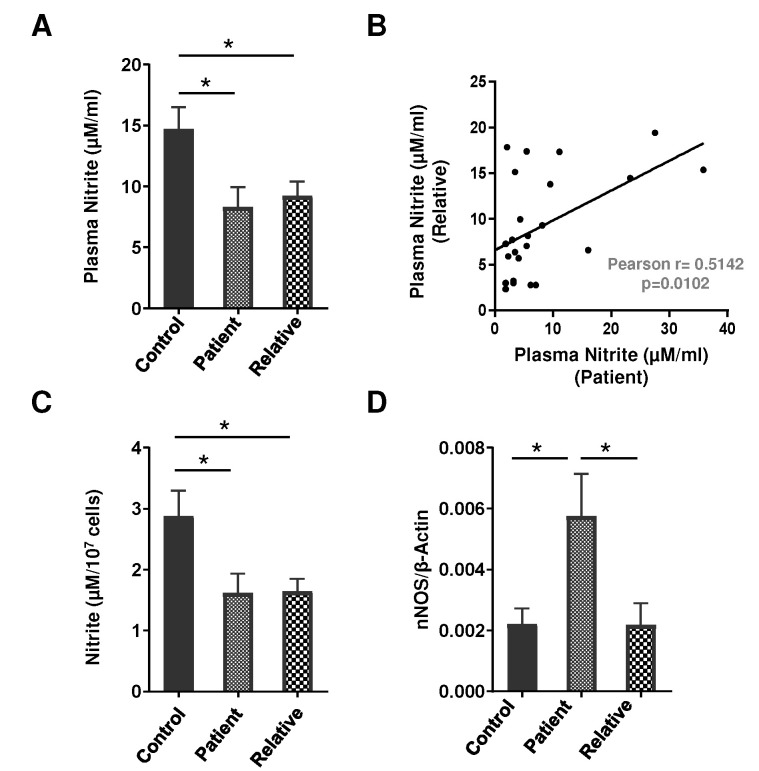
(**A**) Total nitrite content (assessed by a modified Griess assay) in the plasma of drug-naive depression patients (*n* = 27), their first-degree relatives (*n* = 24), and healthy volunteers (*n* = 26). F (2, 74) = 4.953; * *p* < 0.05. (**B**) Correlations in the plasma nitrite content between depressive patients and their first-degree relatives. (**C**) Total nitrite content (assessed by a modified Griess assay, healthy controls *n* = 27, depression patients and first-degree relatives, *n* = 18 in every group) F (2, 60) = 4.422; * *p* < 0.05, and (**D**) nNOS mRNA expression as analyzed by a real-time PCR using β-Actin as an internal loading control in the PMNs of drug-naive depression patients, their first-degree relatives, and healthy volunteers (depression patients *n* = 22, first-degree relatives *n* = 21, healthy controls *n* = 22). F (2, 62) = 4.709; * *p* < 0.05. (**A**,**C**,**D**) Data expressed as mean ± SEM.

The total nitrite content in the plasma was significantly lower in both patients (~0.56 fold, *p* < 0.05, 95% CI: 1.182 to 11.59) and their first-degree relatives (~0.52 fold, *p* < 0.05, 95% CI: 0.1071 to 10.83), as compared to the healthy volunteers. Interestingly, a statistically significant positive correlation in the plasma nitrite content was established between patients and their first-degree relatives (*r* = 0.5142, *p* = 0.0102) (Figure 2A,B). Next, to investigate the regulation of NO, nitrite content and nNOS mRNA expression were assessed in the PMNs of depression patients, their first-degree relatives, and healthy controls. Depressive patients showed a decreased total nitrite content in the PMNs (~0.56 fold, *p* < 0.05, 95% CI: 0.05529 to 2.469), while the expression of the nNOS transcript was significantly more (~2.6 fold, *p* < 0.05, 95% CI: −0.006762 to −0.0003445) in the PMNs of these patients as compared to their first-degree relatives and the healthy volunteers (Figure 2C,D). Similar to plasma nitrite, a positive correlation was observed in the total nitrite in the PMNs between depression patients and first-degree relatives (*r* = 0.6762, *p* = 0.0021; Table 3). Indeed, nNOS isoform mRNA expression was independent of disease severity, HAM-D and GAF score, age, gender, number of episodes, duration of illness, and family history.

### 3.3. Serum Cortisol Level in Depression Patients and First-Degree Relatives

**Figure 3:**
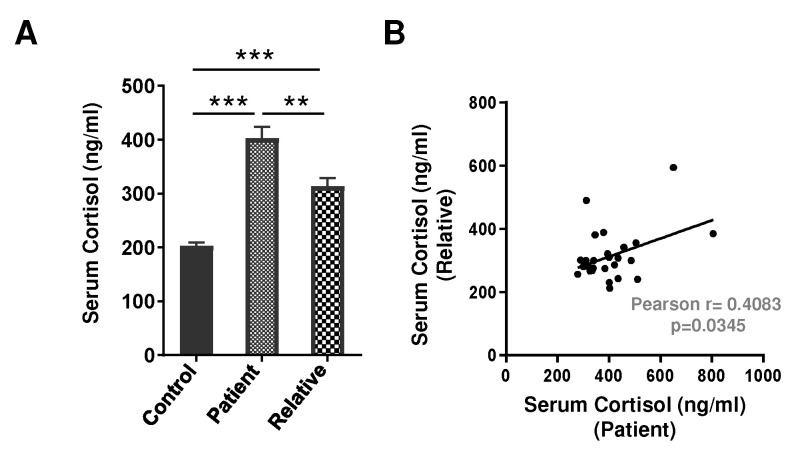
(**A**) Serum cortisol level in drug-naive depression patients (*n* = 29), their first-degree relatives (*n* = 27), and healthy volunteers (*n* = 24). Data expressed as mean ± SEM. F (2, 77) = 36.23; ** *p* < 0.01 and *** *p* < 0.001. (**B**) Correlation in serum cortisol levels between patients and their first-degree relatives.

Since chronic stress raises the incidence level of depression and is highly correlated with cortisol hypersecretion (50), we next analyzed serum cortisol levels for the association between depression patients and their first-degree relatives. The serum cortisol level was significantly elevated in patients (~2-fold, *p* < 0.001, 95% CI: −256.5 to −144.0) as well as their first-degree relatives (~1.5-fold, *p* < 0.001, 95% CI: −168.2 to −53.93), as compared to healthy volunteers (Figure 3A). Our findings further revealed a positive correlation in serum cortisol levels between patients and their first-degree relatives (*r* = 0.4083, *p* = 0.0345), though the level of serum cortisol among the patients was significantly higher than their first-degree relatives (Figure 3B).

## 4. Discussion

Oxidative/nitrosative stress (ROS/RNS) and inflammation are the critical pathological processes of MDD. The results of this study contribute towards a better understanding of the familial association of depressive disorder, and for the first time have demonstrated a positive correlation in neutrophil ROS/RNS, plasma nitrite, and serum cortisol levels between the patients of MDD and their first-degree relatives. The experimental group consisted of 29 patients and 27 first-degree relatives, while the control group was comprised of 27 healthy subjects. As shown in Table 1, the majority of the subjects were young and almost equally distributed in terms of gender, leukocytes count, and other biochemical parameters. Elevated oxidative stress in MDD patients is well established [4,6,7,8,10,13,14,36]; however, the adjoining familial risk factor is not well defined. Parental depression predisposes a resilient risk factor in offspring [51], and Zalar et al. have shown that the genetic make-up of MDD patients with a positive family history of depression is different from sporadic cases [52]. Using a robust and reliable flow cytometry methodology, the present study demonstrated increased ROS/RNS levels in the PMNs of MDD patients. Of note, a study by Frank et al. has shown elevated ROS generation by the monocytes of depressive patients and linked their role with inflammation [53]. To date, most studies have focused on analyzing the oxidative stress in depression patients only and a positive correlation between oxidative stress and severity of depression has been reported [10,12]. For the first time, our study has established a positive association in the ROS/RNS level between major depressive patients and their first-degree relatives and highlighted it as a potential biomarker to estimate risk factors in the family. 

The inflammatory enzyme MPO plays a fundamental role in oxidant production by neutrophils and is linked as a specific marker of microglial immune activation in MDD [41]. By analyzing monozygotic and dizygotic twin pairs, Vaccarino et al. recognized a harmonizing association between MDD and MPO and demonstrated 32% higher levels of serum MPO in twins with prior family history, which increased to the level of 77% among dizygotic MDD-discordant twin pairs [41]. Our findings revealed an elevated MPO activity in the PMNs of both depressive patients and their first-degree relatives, though no correlation in the blood relatives was observed. These observations are synchronized with Talarowska et al. and Gałecki et al., who demonstrated an elevated leukocytes MPO transcript and serum MPO protein in depressive patients [42,54].

A meta-analysis of double-blind, placebo-controlled clinical trials has shown that an antioxidant, N-acetylcysteine (NAC), ameliorates depressive symptoms by modulating the activity of the glutamate transporter, chelating heavy metals, and reducing inflammatory markers [55,56,57]. NAC supplementation also increases cellular cysteine levels and reinforces GSH-related antioxidant defense. Numerous preclinical and clinical studies have further suggested that the consumption of dietary antioxidants such as ascorbic acid, tocopherol, beta-carotene, polyphenols, isoflavonoids, and omega-3 polyunsaturated fatty acids significantly improves depressive symptoms in patients [58,59,60,61]. Indeed, conventional antidepressant medications including doxepin, fluoxetine, and vortioxetine endowed with oxidative and nitrosative scavenging activities have a protective effect [62].

Nitric oxide modulates major neurotransmitters such as norepinephrine, serotonin, dopamine, and glutamate involved in the neurobiology of major depression [19]. This study showed a decreased level of total nitrite content in the plasma of both depressive patients and their first-degree relatives. Furthermore, for the first time, our study revealed a positive correlation in the plasma nitrite between patients of MDD and their first-degree relatives, suggesting an elevated risk of depressive disorder in the blood relatives. These findings persisted regardless of age, gender, family income, fasting blood sugar level, and total leukocyte count. In a recent study, Ali-Sisto et al. evaluated the NO metabolism in patients of MDD and demonstrated lower levels of arginine as well as a lower global arginine bioavailability ratio (GABR) in the serum of MDD patients [63]. Likewise, others have recorded decreased NOS activity (L-Cit/L-Arg) and plasma nitrite levels in major depressive patients [64,65,66]. Of note, antidepressant medications in depressed patients improve NOS activity and predict drug response [66]. Indeed, there are conflicting results in the literature and Suzuki et al. have reported elevated plasma nitrate levels in depressive patients [66]. An additional study by Herken et al. showed no significant change in the level of plasma nitrite in patients with major depression [4]. The major limitation of these studies was their small sample size and the inclusion of patients taking an antidepressant (imipramine, amitriptyline, or mianserin), sedative–hypnotic (nitrazepam or triazolam), and anti-anxiety (etizolam or tandospirone) drugs [67]. Therefore, a direct comparison between drug-naive depression patients with patients taking medication is always deceptive. 

Several clinical studies in post-mortem brains have shown reduced nNOS expression in the hypothalamus and locus coeruleus of depression patients [68,69]. Interestingly, Galecki and colleagues following a large study of depressive patients demonstrated an association between nNOS/iNOS gene polymorphism and increased disease susceptibility [70]. Neuronal NOS is constitutively expressed in resting human neutrophils and plays a central role in NO generation in the PMNs. However, no report is available in the literature that defines the status of nNOS isoforms in the PMNs of depressive patients and their first-degree relatives. We showed a lower level of neutrophil nitrite among patients with major depression compared to controls. This result is consistent with a previous study by Srivastava et al. [7]. Indeed, for the first time, our study further analyzed first-degree relatives and demonstrated that lower neutrophil nitrite levels project an enhanced risk in the family. In addition, this study provides the first data on the mRNA expression of nNOS, which was significantly higher in the PMNs of drug-naive depression patients, but not in first-degree relatives. The increased nNOS transcript but reduced total nitrite level in the PMNs of depression patients might be linked with a lower arginine level and bioavailability as well as decreased NOS activity in depression patients [63,64,65,66]. Van Amsterdam et al. (1999) reported a significant correlation between neopterin and biopterin (N:B) ratios and the severity of depression [22]. A raised N:B ratio implies a failure to convert neopterin to biopterin, which possibly reduces the availability of tetrahydrobiopterin, an essential cofactor for the formation of nitric oxide. Derangements of the neopterin–biopterin pathway could be another reason for finding lower nitric oxide metabolites in patients with depression. Importantly, NOS activity increases significantly over time after antidepressant treatment, and the inhibition of NOS isoforms led to antidepressant-like effects in mice [42,66]. 

The current patient samples showed elevated levels of cortisol in patients as well as in their blood relatives. Serum cortisol is known to be elevated in 20 to 40 percent of depressed patients [3]. However, it is not known if raised levels of glucocorticoids are the cause or effect of depression. It is possible that high cortisol levels do not cause depression but are a consequence of stress. Zunszain et al. have also reported elevated levels of glucocorticoids in patients with MDD [71]. The patients studied also had consistently elevated cortisol levels which could have resulted in a reduction in nitric oxide metabolites in the plasma. It has been reported that this elevated level of cortisol settles with effective treatment [3]. This study also observed increased levels of serum cortisol in first-degree relatives and established a positive correlation with depression patients. There is no study that has assessed the cortisol level in healthy first-degree relatives of patients of depression; however, the implications of these findings in depression pathogenesis need further exploration. The raised level of cortisol in the patients could explain why nitrite levels were low despite increased nNOS mRNA expression. nNOS activity is also inhibited by glucocorticoids [37,72]; multiple mechanisms thus seem to be responsible for the regulation of nitric oxide synthesis and metabolism in MDD. Overall, our results suggest the potential use of nitrite in the plasma/PMNs as the marker of MDD; however, further studies are required to explore the interaction of various regulators in nitric oxide synthesis and the metabolic pathway.

### Limitations

The sample size in the present study was small. It was not possible to match the diet of the subjects in the study, which may affect nitrite content. Subjects using nicotine could not be excluded as this could have limited the sample size. A comparison could not be made between the groups of first-episode depression and recurrent depression due to the small number of subjects in the latter group. Similarly, a comparison could not be made between the parameters of patients with positive and negative histories of mood disorders in blood relatives due to the very small number of subjects in the former group.

## 5. Conclusions

Our findings provide initial support for the family-based association of depressive disorder and have shown that neutrophil ROS/RNS, plasma nitrite, and serum cortisol levels were positively correlated between the patients of MDD and their first-degree relatives and can be used as potential biomarkers to estimate risk factor in the family. Obtaining a family history of depression and its severity and impairment in previous generations might help to identify persons at high risk for psychopathology at a young age. However, further research in larger, more diverse samples of depression patients is needed to extend these observations. 

## Figures and Tables

**Table 1:** Demographic profile of depressive patients, their first-degree relatives, and healthy controls.

	Control	Patient	Relative
Number of patients	27	29	27
Age, Years (mean ± SEM)	29.25 ± 0.90	30.24 ± 1.84	31.11 ± 1.85
Gender (M/F), *n* (%)	18 (66.67%)/9 (33.33%)	13 (44.82%)/16 (55.17%)	15 (55.56%)/12 (44.44%)
FBS, mg/dL (mean ± SEM)	77.25 ± 2.1	81.07 ± 1.77	76.74 ± 2.0
Cholesterol, mg/dL (mean ± SEM)	109.4. ± 2.4	116.9 ± 3.0	117.7 ± 4.5
Total Bilirubin, mg/dL (mean ± SEM)	0.5700 ± 0.024	0.5169 ± 0.0178	0.5833 ± 0.0212
ALP, U/L (mean ± SEM)	120.9 ± 7.7	129 ± 9.2	114.5 ± 9.6
SGPT, U/L (mean ± SEM)	30.25 ± 1.58	29.24 ± 1.92	33.67 ± 1.93
TLC (mean ± SEM)	6554 ± 210.8	6721 ± 209.6	6670 ± 221.5

FBS: Fasting blood sugar, ALP: Alkaline Phosphatase, SGPT: serum glutamic-pyruvic transaminase, TLC: Total leukocyte count.

**Table 2:** Clinical profile of depressive patients.

Variable	
Diagnosis	
Major depressive disorder (1st episode), *n* (%)	25 (86.21%)
Major depressive disorder, recurrent, *n* (%)	04 (13.79%)
Duration of current episode, (mean ± SEM)	3.94 ± 0.5838 months
Family of mood disorder present, *n* (%)	05 (17.24%)
Severity of depression, score on HAM-D, (mean ± SEM)	19.86 ± 0.7636
Functioning, score on GAF, (mean ± SEM)	34.10 ± 1.905

HAM-D: 17-item Hamilton Depression Rating Scale, GAF: Global Assessment of Functioning Scale.

**Table 3:** Correlations between the investigated parameters of depression patients and their first-degree relatives.

	PMNs ROS	Plasma Nitrite	PMNs Nitrite	nNOS mRNA Expression	PMNs MPO	Cortisol
*r*	0.7956	0.5142	0.6762	0.2604	0.01353	0.4083
*p*	0.0001*	0.0102 *	0.0021 **	0.2542	0.9603	0.0345 *
*n*	18	24	18	21	16	27
95% CI	0.5230 to 0.9205	0.1398 to 0.7600	0.3060 to 0.8688	−0.1929 to 0.6222	−0.4854 to 0.5058	0.03354 to 0.6825

*r*, Pearson’s correlation coefficient; *n*, sample size; CI, confidence interval; * *p* < 0.05, ** *p* < 0.01.

## Data Availability

All data used in this study are present in the main text. For original data, please contact the corresponding author.
